# Climate change and its implications on health and the healthcare system: A perspective from Pakistan

**DOI:** 10.1016/j.amsu.2022.104507

**Published:** 2022-08-26

**Authors:** Kainat Riaz, Mohammad Ahmad, Saqib Gul, Mohammed Hamza Bin Abdul Malik, Mohammad Ebad Ur Rehman

**Affiliations:** Department of Medicine, Dow University of Health Sciences, New Labour Colony, Nanakwara, Karachi, Pakistan; Department of Medicine, Hamdard College of Medicine and Dentistry, Hamdard University, Karachi, Pakistan; Department of Medicine, Services Institute of Medical Sciences, Lahore, Pakistan; Department of Medicine, Rawalpindi Medical University, Block E Satellite Town, Rawalpindi, Pakistan

**Keywords:** Climate change, Healthcare, Pakistan

To the Editor,

The global climate crisis is a deadly hazard to our population and has turned out to be a great challenge for global health. The temperature of the Earth has been rising rapidly, with a total increase of 1.8 °F since the late 1800s [1]. This rise in temperature results in a great number of unfortunate events such as droughts, flooding, and wildfires. The intensity of heat waves has increased markedly, making it favorable for dehydration and cold waves have intensified as well [2]. These problems make it difficult for people to survive by causing injuries, mental health disturbances, physical illness, property damages, water contamination as well as deaths [1]. Pakistan is bound to be greatly affected by this crisis due to its geographic location, depending on agriculture, and the weak healthcare system [3]. Being a poor country, it has very few resources to cope with the changing climate.

The recent findings on the impact of Climate change on health in Pakistan are very alarming. A United Nation's report claims that 44% of children suffer from stunted growth primarily because of malnutrition. This is a serious consequence of Pakistan's food insecurity and poverty which is linked to climate change. Unseasonal rains and smog are contributing to viral, waterborne, and vector-borne illnesses like Zika virus, Dengue, Typhoid, and Malaria [4].

In Pakistan, the climate crisis is primarily associated with temperature increases and heat waves, which are expected to cause an increase in the prevalence of respiratory and cardiovascular diseases, and water-borne illnesses [3]. The glacier-fed River Indus and its tributaries provide irrigation for about 90% of the world's agricultural land. The pace of glacier melt has accelerated due to the climate crisis, which will increase the likelihood of glacier lake outburst floods and flash floods downstream. This will have an increasingly negative impact on agriculture-related activities, food production, and livelihoods, as well as the nation's economy [5].

Pakistan could also run out of water by 2040 if the authorities don't take any serious action as Pakistan already ranks third on the list of countries facing acute water shortages according to a 2018 report by the IMF [6]. According to WHO's health country profile for Pakistan, there's a risk of severe flooding in the country due to rising sea levels which may affect up to 1.2 million people by the year 2100. The average temperatures are expected to rise to 6.1° Celsius until that time. This could lead to heat-related deaths to rise, which were recorded as 10 deaths per 100,000 until 1990 but are expected to reach 63 deaths per 100,000 until the year 2080. The elderly, children, and the chronically ill population are especially vulnerable to the expected increase in heat waves. The number of days with extreme rainfall and the number of consecutive dry days are both expected to rise, leading to both flooding and drought in different parts of the country, leading to severe mental and physical health issues coupled with food shortages, putting an enormous burden on healthcare organizations. Amongst vector-borne illnesses, Malaria could pose a threat to around 46 million people annually by the year 2070 but can be controlled drastically by reducing carbon emissions. Outdoor air pollution is a huge contributor for respiratory illnesses and WHO data collected in major cities of Pakistan showed a considerably higher value for the mean PM 2.5 levels recommended by WHO for a clean environment. In addition to that, the Rural population in the country uses Solid fuels like coal etc for cooking which is causing an increase in the total number of deaths from Ischemic heart disease, Stroke, Lung Cancer and COPD particularly in women who engage in household chores and cooking.

What needs to be done is a joint and coordinated effort in improving the infrastructure in the country, coupled with education and a shift toward clean energy. Efficient public transportation systems can play a part in reducing inactivity in the population and hence helping them prevent chronic illnesses. Walking and cycling along with compact urban household planning can help reduce greenhouse emissions and provide a cleaner air quality index in bigger cities.

Pakistan formulated a National Climate change Policy in 2012 and a framework was laid for its implementation in 2014. “Pakistan Vision 2025” is a comprehensive plan which includes positive inputs on the future of sustainable development and economic growth in the country which will help reduce the consequences of Climate Change in Pakistan [7].

## Ethical approval

Not applicable.

## Source funding

None.

## Author contribution

Kainat Riaz: Study conception, write-up, critical review and approval of the final version, Mohammad Ahmad: Study conception, write-up, critical review and approval of the final version, Saqib Gul: Study conception, write-up, critical review and approval of the final version, Mohammed Hamza Bin Abdul Malik: Study conception, write-up, critical review and approval of the final version, Mohammad Ebad Ur Rehman: Study conception, critical review and approval of the final version.

## Trial register number

1. Name of the registry: Not applicable
2. Unique Identifying number or registration ID: Not applicable
3. Hyperlink to your specific registration (must be publicly accessible and will be checked): Not applicable

## Guarantor

Kainat Riaz.

Mohammad Ebad Ur Rehman.

## Consent

Not applicable.

## Declaration of competing interest

The authors have no conflicts of interest.
